# Identifying the metabolomic fingerprint of high and low flavonoid consumers

**DOI:** 10.1017/jns.2017.27

**Published:** 2017-07-14

**Authors:** Kerry L. Ivey, Eric B. Rimm, Peter Kraft, Clary B. Clish, Aedin Cassidy, Jonathan Hodgson, Kevin Croft, Brian Wolpin, Liming Liang

**Affiliations:** 1 Department of Nutrition, Harvard T. H. Chan School of Public Health, Boston, MA, USA; 2 Department of Epidemiology, Harvard T. H. Chan School of Public Health, Boston, MA, USA; 3 Channing Division of Network Medicine, Department of Medicine, Brigham and Women's Hospital and Harvard Medical School, Boston, MA, USA; 4 Department of Biostatistics, Harvard T. H. Chan School of Public Health, Boston, MA, USA; 5 Broad Institute of the Massachusetts Institute of Technology, Boston, MA, USA; 6 Department of Nutrition, Norwich Medical School, University of East Anglia, Norwich, Norfolk, UK; 7 University of Western Australia, School of Medicine and Pharmacology, Perth, WA, Australia; 8 Department of Medical Oncology, Dana-Farber Cancer Institute, Harvard Medical School, Boston, MA, USA

**Keywords:** Flavonoids, Metabolomics, Epidemiology, Population, Diet, HPFS, Health Professionals Follow-Up Study, NHS, Nurses’ Health Study, QC, quality control

## Abstract

High flavonoid consumption can improve vascular health. Exploring flavonoid–metabolome relationships in population-based settings is challenging, as: (i) there are numerous confounders of the flavonoid–metabolome relationship; and (ii) the set of dependent metabolite variables are inter-related, highly variable and multidimensional. The Metabolite Fingerprint Score has been developed as a means of approaching such data. This study aims to compare its performance with that of more traditional methods, in identifying the metabolomic fingerprint of high and low flavonoid consumers. This study did not aim to identify biomarkers of intake, but rather to explore how systemic metabolism differs in high and low flavonoid consumers. Using liquid chromatography–tandem MS, 174 circulating plasma metabolites were profiled in 584 men and women who had complete flavonoid intake assessment. Participants were randomised to one of two datasets: (a) training dataset, to determine the models for the discrimination variables (*n* 399); and (b) validation dataset, to test the capacity of the variables to differentiate higher from lower total flavonoid consumers (*n* 185). The stepwise and full canonical variables did not discriminate in the validation dataset. The Metabolite Fingerprint Score successfully identified a unique pattern of metabolites that discriminated high from low flavonoid consumers in the validation dataset in a multivariate-adjusted setting, and provides insight into the relationship of flavonoids with systemic lipid metabolism. Given increasing use of metabolomics data in dietary association studies, and the difficulty in validating findings using untargeted metabolomics, this paper is of timely importance to the field of nutrition. However, further validation studies are required.

Metabolomics is the study of the complement of metabolites present in biological samples^(^^1^^,^^2^^)^. Application of metabolomic technology to targeted mechanistic investigations has elucidated many mechanisms underlying metabolic and disease pathways^(^^3^^–^^6^^)^. With technological improvements, metabolomic analyses of human samples have now extended to exploratory, population-based, untargeted analyses, where the aim is to identify new or novel biomarkers of disease. Such analyses are typically conducted in well-designed, nested case–control and cohort studies^(^^7^^,^^8^^)^.

With increasing availability of metabolome data in large population-based studies with measures of habitual dietary intake, focus has shifted towards identifying metabolic profiles that are associated with different human exposures, such as diet and lifestyle^(^^9^^,^^10^^)^. Flavonoids are biologically active compounds found in many different foods such as blueberries, tea, wine and chocolate^(^^11^^,^^12^^)^. Given the short half-life of the parent flavonoid compounds^(^^13^^,^^14^^)^, and their complex metabolism within the gastrointestinal tract^(^^15^^–^^17^^)^, there are no validated and reliable biomarkers of total flavonoid intake. Furthermore, although having demonstrated vascular benefits, such as improved nitric oxide homeostasis and endothelial function^(^^18^^–^^20^^)^, the mechanisms underlying the biological effects of flavonoids are complex and yet to be fully elucidated. As such, flavonoids represent an ideal candidate for application to untargeted exposure–metabolome analyses, where flavonoid intake is the independent variable, and the metabolites, the dependent variables, with the aim of identifying how systemic metabolism differs in high and low flavonoid consumers.

Unlike previous flavonoid–metabolome studies^(^^21^^–^^24^^)^, this project did not aim, nor did it have the capacity, to identify biomarkers of flavonoid intake or measure acute effects of flavonoid consumption. In fact, flavonoid compounds were not identified in the analytic platforms used in this study. Instead we aimed to describe the long-term metabolic profiles characteristic of those with habitual high or low total flavonoid intake. With this analysis comes the unique challenge of integrating multiple confounding variables into models where the dependent variables consist of many inter-related metabolite concentrations. The non-independence of the dependent metabolite variables makes the translation of results from metabolome-wide association studies challenging. Canonical discriminant analysis is a far more suitable modelling approach for such studies as it is capable of reducing the dimensionality of the rich dependent data matrix into a linear combination of the metabolite variables that summarise the metabolite variation between different levels of flavonoid intake.

Although reducing the dimensionality of the data, full canonical discriminant analysis incorporates information from all the dependent metabolite variables. Therefore, this analytical approach is not useful in identifying which metabolites are most important in discriminating differing levels of exposure in a population. By applying stepwise feature selection to canonical modelling we can identify the combination of metabolites that best discriminate people with differing flavonoid intake levels. However, the statistical properties of stepwise regression often result in biased parameter estimates.

There is large measurement error in metabolomic assessment, with peak area CV reaching as high as 25 %^(^^25^^)^. As such, metabolome data are better suited to ranking analyses and for discriminating samples with very low and very high metabolite concentrations. Conversely, due to the random error and the large number of parameters innately present in metabolomic datasets, metabolome data are less appropriate for complex model development where each metabolite is assigned a coefficient or weighting variable, such as canonical discriminant analysis. As a result of this over-fitting, we hypothesise that canonical variables created in one cohort (a training cohort) will perform poorly, in terms of discriminatory capacity, when applied to a validation cohort.

We instead put forth that a model based on identifying relative patterns of distinguishing metabolites, and collectively summarising the groups of metabolites that are relatively more or less abundant in individuals with different levels of total flavonoid intake. We term this summary variable the Metabolite Fingerprint Score, and we hypothesise that by identifying biological meaningful patterns that may be subtle, and ignoring the ‘noise’ in the data, the Metabolite Fingerprint Score developed in a training cohort will be able to differentiate high from low flavonoid consumers in a validation cohort. It is the aim of this study to compare the performance of metabolome-wide association studies, full canonical variables, step-wise canonical variables and the Metabolite Fingerprint Score in identifying the metabolomic fingerprint of high and low flavonoid consumers.

## Methods

### Participants

The Health Professionals Follow-Up Study (HPFS) was initiated in 1986 when 51 529 US men 40–75 years working in health professions completed a mailed biennial questionnaire^(^^26^^)^. The Nurses’ Health Study (NHS) was established in 1976 when 121 700 female nurses aged 30–55 years completed a mailed biennial questionnaire^(^^27^^)^.

For this analysis we used data from a nested case–control study exploring the plasma metabolite profile associated with risk of pancreatic cancer^(^^28^^)^: HPFS (*n* 237); NHS (*n* 370). Participants were selected for this study if they had incident pancreatic adenocarcinoma cases diagnosed after blood collection up to 2010 with available plasma and no prior history of cancer. For each case, we randomly selected two controls, matching on cohort, sex, year of birth, smoking status and fasting status (<8 h, ≥8 h). To reduce the influence of subclinical malignancy on plasma metabolite levels, we excluded cases diagnosed within 2 years of blood collection.

**Fig. 1.**
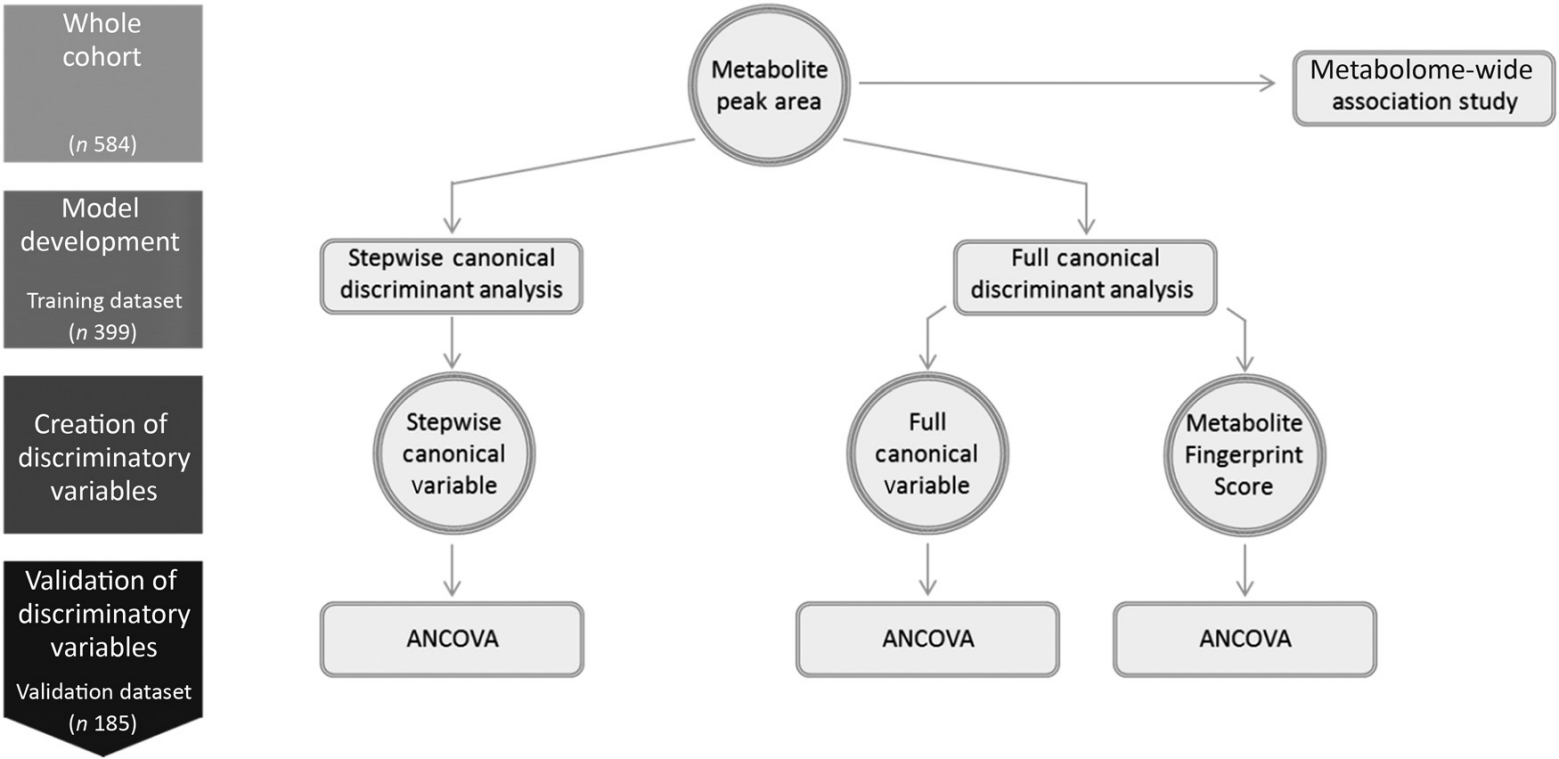
Study design.

The final analysis included 584 participants (192 cases and 392 controls), as they had complete dietary flavonoid intake assessment in conjunction with plasma metabolic profiling, at the same time point. The study was approved by the Human Research Committee at Brigham and Women's Hospital (Boston, MA, USA), and all participants provided consent. For the study design, see Fig. 1.

### Dietary intake assessment

In 1994 and 1990, the HPFS and NHS participants, respectively, completed a validated FFQ to determine habitual intake of foods and beverages over the preceding year. In particular, participants reported how often, on average, they consumed each food of a standard portion size. Based on the FFQ responses, as well as a comprehensive flavonoid food composition database, habitual daily total flavonoid intake was estimated using standard methods^(^^29^^)^. Specifically, intake was computed by multiplying the frequency of consumption for a particular portion size by the flavonoid content in that particular food item and then summing the product across all food items. Total flavonoid intake was derived by summing up the five major flavonoid subclasses evaluated in the current analysis: flavonols; flavones; flavanones; flavan-3-ols (including both monomers and polymers); and anthocyanins. Isoflavones were not included in this analysis as they are almost exclusively associated with soya intake, with daily intakes less than 3 mg/d in this population of US adults.

### Plasma samples

Blood samples in EDTA tubes were collected in the HPFS from 1993 to 1995, and in heparin tubes for the women in the NHS from 1989 to 1990. Blood samples were collected by participants, mailed overnight on cold packs, and then spun to collect and store plasma (delayed processing)^(^^30^^,^^31^^)^.

### Metabolite profiling

Plasma metabolites were measured as peak areas by a liquid chromatography–MS metabolomics platform directed by C. B. C. at the Broad Institute of the Massachusetts Institute of Technology and Harvard University (Cambridge, MA, USA). Metabolite profiling methods were developed using reference standards of metabolites to determine chromatographic retention times, MS multiple reaction monitoring transitions, declustering potentials and collision energies^(^^7^^)^. We evaluated plasma from ten volunteers with plasma collected simultaneously in heparin and EDTA tubes. For the branched-chain amino acids, Spearman correlation coefficients between heparin and EDTA samples were 0·85 for isoleucine, 0·88 for leucine and 0·95 for valine.

In prior pilot work^(^^25^^)^, we determined that thirty-two metabolites had poor reproducibility in samples with delayed processing, so these metabolites were excluded as they could not be reliably measured in two of the participating cohorts. In the present study, three heparin plasma pools (fifty-seven total quality control (QC) samples) and three EDTA plasma pools (128 total QC samples) were randomly interspersed among participant samples as blinded QC samples.

This study included only identified peaks generated from each untargeted platform. We calculated mean CV for each metabolite across QC plasma pools and set an *a priori* threshold of ≤25 % for satisfactory reproducibility. Based on this criterion, twenty-two metabolites with mean CV > 25 % were excluded from our analyses. Five metabolites had undetectable levels for >10 % of participants and were also excluded (Supplementary Appendix S1). For other metabolites with ≤10 % undetectable, zero values were recoded to the minimum peak area value for each individual metabolite. A total of 174 metabolites passed QC and were included in the analysis (Supplementary Appendix S2).

### Statistical analysis

Prior to analysis all metabolite peak areas were normalised by means of log transformation. Flavonoid intake was divided into tertiles, and metabolites were normalised (mean: 0, standard deviation: ±1) in both the HPFS and NHS cohorts. All data were then merged and participants were randomised to either the training dataset or the validation dataset in a 2 to 1 fashion. To ensure all metabolites have the opportunity to contribute equally to the canonical models, the values were also normalised after randomisation into the training and validation datasets. All analyses were performed with the SAS 9.2 statistical package (SAS Institute, Inc.).

The multivariate-adjusted models included case/control status, cohort, quintiles of energy intake, smoking status, age at blood collection, the Alternative Healthy Eating Index (minus alcohol) score and alcohol consumption.

### Metabolome-wide association study

We conducted both unadjusted and multivariate-adjusted Spearman correlations between flavonoid intake and each of the 174 metabolites, in individual models. Using a Bonferroni correction for multiple hypothesis testing^(^^32^^)^, only those metabolites with *P* ≤ 0·0003 (0·05/174) were considered to be statistically significantly associated with total flavonoid intake.

### Development of discriminatory variables in the training dataset

The stepwise canonical variable was computed from the canonical coefficients of a stepwise canonical correlation analysis. The independent variable was high (highest tertile) or low (lowest tertile) of flavonoid consumption (moderate flavonoid consumers were excluded from this analysis) and the dependent variable matrix consisted of all 174 metabolites. The significance level for adding variables in the forward selection mode, or removing them in the backward elimination mode, was 0·15. No metabolites were forced into the model.

In a full canonical discriminant analysis, the full canonical variable was computed from the canonical coefficients from the principal components analysis eigenvector encapsulating the greatest degree of discrimination. As such, all 174 metabolites contribute to the computation of the full canonical variable. The Metabolite Fingerprint Score is calculated as follows:
1
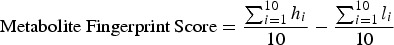

where *h* is the peak area of metabolites with the highest canonical discriminant coefficient, and *l* is the peak area of metabolites with the lowest canonical discriminant coefficient. Variables included in the Flavonoid Metabolite Fingerprint Score computation are shown in Table 1.

**Table 1.** Variables included in the Flavonoid Metabolite Fingerprint Score computation

Metabolites with the highest canonical coefficients	Metabolites with the lowest canonical coefficients
*h*_1_ = Lysophosphatidylcholine C18 : 0	*l*_1_ = Lysophosphatidylethanolamine C22 : 6
*h*_2_ = TAG C54 : 6	*l*_2_ = Diacylglycerol C34 : 2
*h*_3_ = TAG C50 : 4	*l*_3_ = TAG C50 : 0
*h*_4_ = Sphingomyelin C18 : 1	*l*_4_ = TAG C54 : 4
*h*_5_ = Diacylglycerol C36 : 2	*l*_5_ = Lysophosphatidylcholine C20 : 3
*h*_6_ = Phosphatidylcholine C34 : 4	*l*_6_ = Sphingomyelin C18 : 0
*h*_7_ = TAG C54 : 1	*l*_7_ = TAG C48 : 2
*h*_8_ = TAG C56 : 7	*l*_8_ = TAG C52 : 1
*h*_9_ = TAG C48 : 1	*l*_9_ = TAG C52 : 6
*h*_10_ = TAG C50 : 1	*l*_10_ = Lysophosphatidylcholine C14 : 0

As outlined in Equation 1, the Metabolite Fingerprint Score incorporated the ten metabolites with the largest negative and positive canonical discriminant coefficients, identified by the full canonical discriminant analysis (described above). To explore the influence that fasting status and dietary fat intake have on the discriminatory capacity of the Metabolite Fingerprint Score, we repeated the multivariate-adjusted ANCOVA model, with additional adjustment for fasting status as well as intakes of *trans*-fat, cholesterol, saturated fat, monounsaturated fat and polyunsaturated fat. Model 1 excluded the Alternate Health Eating Index, and model 2 included the Alternate Health Eating Index. An additional sensitivity analysis was conducted to explore the relationship of fasting status and dietary fat intake with the Metabolite Fingerprint Score, via unadjusted and multivariate-adjusted (partial) Spearman correlation and ANCOVA models.

### Evaluating the performance of the discriminatory variables in the validation cohort

Using the models developed in the training dataset, the three discriminatory variables (stepwise canonical variable, full canonical variable, and Metabolite Fingerprint Score) were computed in the validation dataset participants. We then performed separate unadjusted and multivariate-adjusted ANCOVA for each discriminatory variable, with tertiles of flavonoid intake as the independent variable.

## Results

### Cohort characteristics

The characteristics of the study population are presented in Table 2. The mean daily flavonoid intake was 344 (sd 310) mg/d, with high total flavonoid consumers having an over 5-fold greater mean total flavonoid intake than low total flavonoid consumers. Moderate and high total flavonoid consumers were less likely to have a history of smoking, and moderate consumers were older at the time of blood draw. Following random dichotomisation of the whole cohort, the test and validation cohorts were well matched in terms of total flavonoid intake, as well as other baseline characteristics.

**Table 2.** Cohort characteristics stratified by level of flavonoid consumption (Mean values and standard deviations; numbers and percentages)

	Low intake (*n* 183)				Moderate intake (*n* 214)				High intake (*n* 187)			
	Mean	sd	*n*	%	Mean	sd	*n*	%	Mean	sd	*n*	%
Flavonoid intake (mg/d)	130	38	–	–	253	44	–	–	659	379	–	–
Pancreatic cancer cases	–	–	57	31	–	–	70	33	–	–	65	35
Age at blood draw (years)	62	8	–	–	63	7	–	–	62	7	–	–
Males	–	–	73	40	–	–	77	36	–	–	71	38
Smoking status												
Never	–	–	52	28	–	–	96	45	–	–	84	45
Previous	–	–	90	49	–	–	98	46	–	–	90	48
Current	–	–	41	22	–	–	20	9	–	–	13	7
Energy intake												
kJ/d	7828	2443	–	–	8381	2536	–	–	7607	2050	–	–
kcal/d	1871	584	–	–	2003	606	–	–	1818	490	–	–
AHEI (index score)	51	10	–	–	56	11	–	–	56	11	–	–

AHEI, Alternative Healthy Eating Index.

### Metabolome-wide association study in the whole cohort

**Fig. 2.**
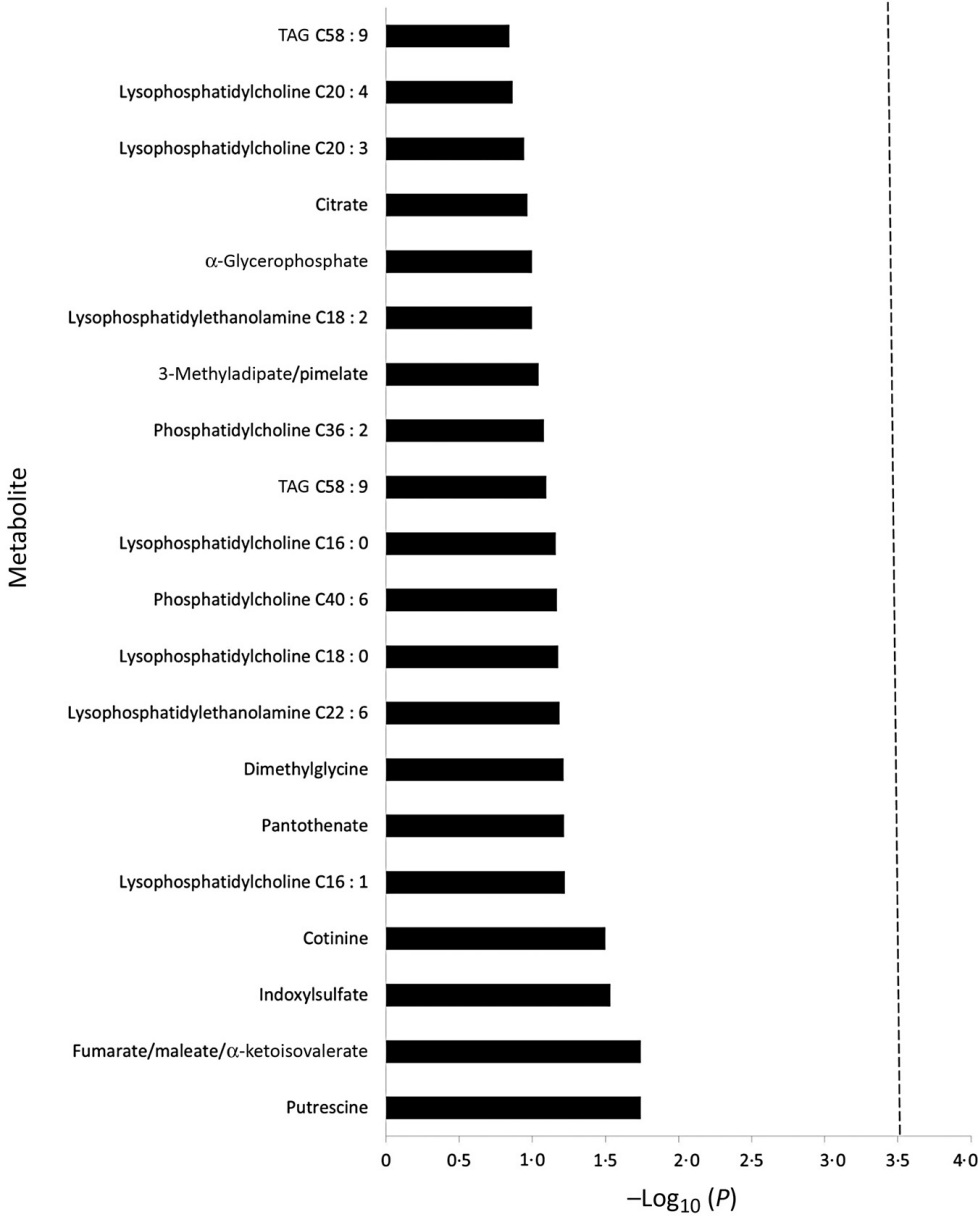
Multivariate-adjusted metabolome-wide association study of flavonoid intake and the twenty metabolites to which it is most strongly associated. -----, Bonferroni-corrected level of significance required, after accounting for 174 multiple comparisons. Multivariate-adjusted model includes case/control status, cohort, quintiles of energy intake, smoking status, age at blood collection, the Alternative Healthy Eating Index (minus alcohol) score and alcohol consumption.

Using the significance cut-off of *P* ≤ 0·0003, in unadjusted analysis, the only metabolite significantly associated with flavonoid intake was cotinine; a metabolite of nicotine and a biomarker of tobacco smoke exposure^(^^33^^)^ (Spearman correlation coefficient −0·153; *P* = 0·0002). However, this relationship did not remain in the partial Spearman rank analysis which controlled for the variables in the multivariate models (Fig. 2).

We then repeated the partial Spearman rank analysis, with cigarette smoking as the independent variable, and controlled for flavonoid intake as well as the multivariate variables, and observed a strong positive association of cigarette smoking with cotinine concentration (*P* < 0·0001). This result highlights the importance of confounders in exposure–metabolome association studies, and, by observing the established causal association between cigarette exposure and cotinine concentrations, it validates our capacity to observe biologically meaningful exposure–metabolome associations in this cohort.

In the flavonoid–metabolome partial Spearman correlation analysis, no metabolites were significantly associated with flavonoid intake (Fig. 2).

### Development of discriminatory variables in the training dataset

The training dataset was used to compute three discriminatory variables: (1) stepwise canonical variable; (2) full canonical variable; and (3) Metabolite Fingerprint Score.

### Computing the stepwise canonical variable

`The forwards–backwards stepwise canonical analysis entered twenty-eight metabolites into the model, four of which were removed. The final model included the following metabolites: cotinine; 4-pyridoxate; thiamine; indoxylsulfate; isocitrate; β-hydoxybutyrate; aconitate; putrescine; sorbitol; glutamate; dimethylglycine; hydroxyphenylacetate; aminoisobutyric acid; quinolinate; sphingomyelin C16 : 0; TAG C48 : 1; TAG C56 : 2; TAG C54 : 8; TAG C50 : 4; TAG C50 : 0; cholesteryl ester C18 : 3; cholesteryl ester C20 : 5; phosphatidylcholine C32 : 2; diacylglycerol C34 : 2.

In the training dataset, the stepwise canonical variable was able to distinguish moderate and high flavonoid consumers from low flavonoid consumers (Table 3).

**Table 3.** ANCOVA of flavonoid discrimination variables by flavonoid intake group in the training dataset (Least squared mean values with their standard errors)

	Low intake (*n* 123)		Moderate intake (*n* 147)		High intake (*n* 129)	
	Least squared mean	sem	Least squared mean	sem	Least squared mean	sem
Stepwise canonical variable†						
Unadjusted	−0·77	0·10	−0·03*	0·09	0·77*	0·10
Multivariate-adjusted‡	−0·88	0·13	−0·17*	0·14	0·57*	0·15
Full canonical variable†						
Unadjusted	−1·86	0·18	0·25*	0·17	1·50*	0·18
Multivariate-adjusted‡	−1·76	0·24	0·21*	0·25	1·53*	0·26

* Significantly different from low consumers (*P* < 0·05).

† Canonical value.

‡ Multivariate-adjusted model includes case/control status, cohort, quintiles of energy intake, smoking status, age at blood collection, the Alternative Healthy Eating Index (minus alcohol) score and alcohol consumption.

### Computing the full canonical variable

The greatest degree of discrimination between low and high flavonoid consumers was observed along eigenvector 1 of the full canonical discriminant analysis, and therefore, full canonical variable 1 was used for all subsequent analyses.

In the training dataset, the full canonical variable was able to distinguish moderate and high flavonoid consumers from low flavonoid consumers (Table 3).

### Computing the Metabolite Fingerprint Score

The Metabolite Fingerprint Score was computed using the results of the full canonical model to dictate which metabolites are included in the computation (Equation 1). The ten ‘high’ and ten ‘low’ metabolites contributing to the Metabolite Fingerprint Score computation were all lipid metabolites. There were six TAG metabolites in the ‘high’ group, and five in the ‘low’ group. The other metabolite groups contributing to both the ‘high’ and ‘low’ metabolite groups were sphingomyelins, diacylglycerols and lysophosphatidylcholines. Phosphatidylcholine was unique to the ‘high’ metabolite group, and lysophosphatidylethanolamine was unique to the ‘low’ metabolite group.

**Fig. 3.**
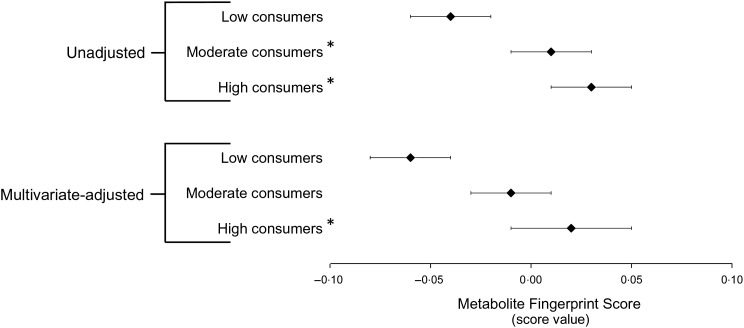
ANCOVA of the Metabolite Fingerprint Score by flavonoid intake group in the training dataset. Results are least squared mean values, with their standard errors represented by horizontal bars. * Significantly different from low consumers (*P* < 0·05). The multivariate-adjusted model includes case/control status, cohort, quintiles of energy intake, smoking status, age at blood collection, the Alternative Healthy Eating Index (minus alcohol) score and alcohol consumption. Low intake, *n* 123; moderate intake, *n* 147; high intake, *n* 129.

In the training dataset, the Metabolite Fingerprint Score was able to distinguish high flavonoid consumers from low flavonoid consumers (*P* < 0·05) (Fig. 3).

### Assessing the performance of the discriminatory variables in the validation dataset

After developing the three discriminatory variables in the training dataset, we then sought to test their performance in the validation dataset. We were also interested in exploring the effect of multivariate adjustment on the capacity of the discriminatory variables to discriminate high from low flavonoid consumers.

There was no difference in the value of the stepwise canonical variable when comparing high flavonoid consumers with low flavonoid consumers, in either unadjusted or multivariate-adjusted models. Similarly, no discrimination was provided by the full canonical variable in either unadjusted or multivariate-adjusted models (Table 4).

**Table 4.** ANCOVA of stepwise canonical variable and full canonical variable by flavonoid intake group in the validation dataset* (Least squared mean values with their standard errors)

	Low intake (*n* 60)		Moderate intake (*n* 67)		High intake (*n* 58)	
	Least squared mean	sem	Least squared mean	sem	Least squared mean	sem
Stepwise canonical variable†						
Unadjusted	−0·12	0·19	0·02	0·18	0·10	0·20
Multivariate-adjusted‡	−0·51	0·26	−0·51	0·30	−0·56	0·30
Full canonical variable†						
Unadjusted	0·30	0·49	−0·09	0·47	−0·21	0·50
Multivariate-adjusted‡	−1·11	0·67	−1·34	0·78	−1·54	0·80

* No values were significantly different from low consumers (*P* < 0·05).

† Canonical value.

‡ Multivariate-adjusted model includes case/control status, cohort, quintiles of energy intake, smoking status, age at blood collection, the Alternative Healthy Eating Index (minus alcohol) score and alcohol consumption.

**Fig. 4.**
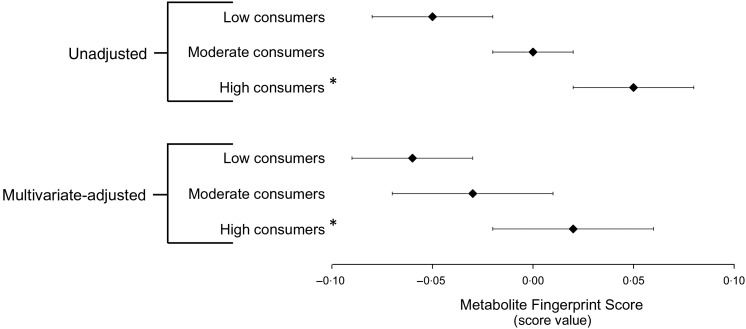
ANCOVA of the Metabolite Fingerprint Score by flavonoid intake group in the validation dataset. Results are least squared mean values, with their standard errors represented by horizontal bars. * Significantly different from low consumers (*P* < 0·05). The multivariate-adjusted model includes case/control status, cohort, quintiles of energy intake, smoking status, age at blood collection, the Alternative Healthy Eating Index (minus alcohol) score and alcohol consumption. Low intake, *n* 60; moderate intake, *n* 67; high intake, *n* 58.

Conversely, when compared with low flavonoid consumers, the value of the Metabolite Fingerprint Score was higher in high flavonoid consumers. This discriminant capacity was evident in both unadjusted and multivariate-adjusted models (*P* < 0·05) (Fig. 4).

### Exploring the lipid nature of the Metabolite Fingerprint Score

As can be seen in Equation 1, the Metabolite Fingerprint Score is largely comprised of lipid metabolites, predominantly TAG, which appear on both the high and the low metabolite lists. To explore the potential role of dietary fat intake in contributing to this pattern, we repeated the validation analysis, adjusting for the variables in the multivariate-adjusted model (excluding Alternate Health Eating Index), with additional adjustment for intakes of *trans*-fat, cholesterol, saturated fat, monounsaturated fat and polyunsaturated fat. This did not influence the significance or strength of discrimination (multivariate adjusted least squared means of low and high consumers: −0·09 (sem 0·04) and +0·01 (sem 0·04), respectively; *P* = 0·0185). Furthermore, none of the dietary fats contributed significantly to the model (*P* > 0·05). Results were similar for the models which also included the Alternate Healthy Eating Index. Furthermore, discrimination capacity was not altered when fasting status was included in the multivariate-adjusted dietary fat model (multivariate adjusted least squared means of low and high consumers: −0·09 (sem 0·04) and +0·01 (sem 0·04), respectively; *P* = 0·0161).

We then sought to explore the independent association of dietary fat intake with the Metabolite Fingerprint Score. None of the dietary fats, nor fasting status, was correlated with the Metabolite Fingerprint Score in either unadjusted or partial (multivariate-adjusted) Spearman correlation models (multivariate-adjusted ANCOVA, *P* > 0·05). Furthermore, for each dietary fat, the Metabolite Fingerprint Score did not discriminate high from low consumers. This was also the case for fasting status, where the Metabolite Fingerprint Score was not significantly different in participants who did and did not fast (multivariate-adjusted ANCOVA, *P* > 0·05).

### Exploring the Metabolite Fingerprint Score performance in the validation dataset

As the Metabolite Fingerprint Score is conceptually and statistically the dependent variable in these analyses, we are not concerned with whether resultant models explain the Metabolic Fingerprint Score, but rather whether the Metabolic Fingerprint Score is different in high and low flavonoid consumers, in the context of a multivariate setting. As such, to determine if the score consisting of ten high and ten low metabolites provides the greatest discrimination of high and low flavonoid consumers, we compared the *F* statistic assigned to the flavonoid consumption variable (high *v.* low intake) in a multivariate-adjusted ANCOVA. This was done for Metabolite Fingerprint Scores comprised of between five and fifteen metabolites, with the null hypothesis that in a multivariate-adjusted setting, the mean Metabolic Fingerprint Score of high and low flavonoid consumers would be the same.

The value of the *F* statistic ranged from 1·51 for the Metabolite Fingerprint Score computed from five high and five low metabolites, to 0·07 for the Metabolite Fingerprint Score computed from fifteen high and fifteen low metabolites. With an *F* statistic of 4·75, well above the critical threshold of 3·9, the Metabolite Fingerprint Score computed from ten high and ten low metabolites had the greatest discriminatory capacity.

## Discussion

Using models created in a training dataset, when applied to a separate validation dataset containing different participants, the Metabolite Fingerprint Score was able to distinguish high from low flavonoid consumers, even after taking into account potential confounders of the flavonoid–metabolome relationship. Conversely, both the stepwise canonical variable and the full canonical variable did not distinguish high from low flavonoid consumers in the validation dataset. In addition, the multivariate-adjusted metabolome-wide association study failed to identify any significant metabolites.

The unadjusted metabolome-wide association study identified a significant, inverse, association between flavonoid intake and cotinine, a biomarker for cigarette smoking^(^^33^^)^. This spurious association manifested because of a strong positive association between cigarette smoking and cotinine peak area, and a strong inverse association between flavonoid intake and smoking status. This association disappeared following multivariate-adjustment, which included adjusting for smoking status, thus highlighting the importance of multivariate adjustment in diet–metabolome studies. Consistent with our hypothesis, the multivariate-adjusted metabolome-wide association study failed to identify any metabolites that were significantly associated with flavonoid consumption. As such, we pursued a pattern-based approach to analysing the data.

The Metabolite Fingerprint Score incorporated a set of ten ‘high’ metabolites that were in higher concentrations in high flavonoid consumers, and lower concentrations in low flavonoid consumers. An additional set of ten different metabolites were also incorporated into the Metabolite Fingerprint Score computation. These ‘low’ metabolites represented those that were in lower concentrations in high flavonoid consumers, and higher concentrations in low flavonoid consumers. This pattern consisting of ten ‘high’ and ten ‘low’ metabolites was found to provide the greatest degree of differentiation in the validation dataset.

This study utilised data from both lipid-soluble and polar metabolomic platforms. The stepwise and full canonical variables incorporated both lipid-soluble and polar metabolites, many of which included metabolites with large CV. Conversely, the Metabolite Fingerprint Score included only lipid metabolites that had a low degree of random measurement error (mean CV of metabolites included in the Metabolite Fingerprint Score: 9·6 %). If metabolites, or their pattern, are biologically related to flavonoid intake, these associations are more likely to be picked up in metabolites which can be assessed with a high degree of precision. As such, the preferential incorporation of low CV metabolites into the score was not by design, but rather because of the properties of the Metabolite Fingerprint Score itself.

Due to the dominance of lipid metabolites in the Metabolite Fingerprint Score, we thoroughly explored the role of dietary fat intake in influencing observed results. The inclusion of fasting status as well as intakes of cholesterol, *trans*-fat, saturated fat, unsaturated fat and polyunsaturated fat into the multivariate-adjusted model did not influence the discriminatory capacity of the Metabolite Fingerprint Score. Furthermore, there was no independent association of fasting status or any the dietary fats with the Metabolite Fingerprint Score. This is not surprising given the equal contribution of lipids to both the ‘high’, and the ‘low’, metabolite groups. These results suggest that dietary fat intake is not driving the observed results, and that the composition of the Metabolite Fingerprint Score may instead be reflecting the underlying differences in fatty acid metabolism amongst high and low flavonoid consumers. It is important to note that the lipid metabolite profile provides information on how lipids are metabolised and created systemically, which is quite distinct from the clinical relationship of flavonoids with total cholesterol, LDL-cholesterol, HDL-cholesterol and total TAG. Although there is a great degree of heterogeneity across studies, there is evidence to suggest possible effects of specific flavonoid compounds on some clinical lipid measurements^(^^34^^)^. Further studies are warranted to fully elucidate the effect that flavonoids have on systemic lipid metabolism.

While it is beyond the scope of this paper to relate the lipid metabolite compounds to potential bioactivity, and the biological interpretation of results arising from lipid metabolomics platforms can be challenging, there are numerous reasons why one might expect the Metabolite Fingerprint Score to be predominated by lipids. It is hypothesised that due to humans’ capacity for fatty acid storage^(^^35^^)^, lipid-soluble metabolites may be a better indicator of long-term alterations in metabolism, such as those explored in this study, when compared with the polar metabolites which often have a high systemic turnover rate. However, the most likely explanation comes from the nature of the metabolome data themselves. With a mean CV of 11·6 % for the lipid-soluble metabolites, and 19·4 % for the polar metabolites, our capacity to reproducibly assess metabolites was much greater for lipid-soluble metabolites in comparison with their water-soluble counterparts.

The Metabolite Fingerprint Score has identified a pattern of two sets of ten metabolites that, in relation to the metabolome as a whole, were in higher and lower concentrations in high flavonoid consumers, when compared with their low flavonoid-consuming counterparts. Furthermore, in a separate validation dataset, we were able to confirm that this pattern of metabolites characterised high from low flavonoid consumers, in individuals distinct from those upon which the score was created. This internal validation of the Metabolite Fingerprint Score is an important first step in understanding the global metabolic pathways affected by high flavonoid consumption. However, unlike polar metabolites which are well described in many common metabolic pathway analysis tools and databases, the role of fatty acids in systemic metabolism is less well characterised. As such, the results of population-based studies such as these are most useful in identifying candidate compounds which can then be further scrutinised in clinical trial settings, as well as by the use of more targeted spectroscopic methods, such as NMR, where the precise fatty acid structure can be scrutinised, and biological significance of this pattern can be unravelled.

Previous diet–plasma metabolome studies, using various study designs, have identified diet–metabolome relationships using a variety of analytical methods; ranging from correlation analysis to cluster analysis^(^^36^^,^^37^^)^. However, it remains uncertain which is the optimal statistical method to apply to diet–metabolome analyses. Despite all three discrimination variables discriminating high from low flavonoid consumers in the dataset in which the models were developed, only the Metabolite Fingerprint Score discriminated high from low flavonoid consumers in the validation dataset. By weighting all included metabolites equally, and incorporating only directionality into the model, the Metabolite Fingerprint Score was able to avoid carrying forward artifacts, or unintended patterns, from the training dataset.

In conclusion, the Metabolite Fingerprint Score was the most valid means of identifying a pattern of inter-related metabolites that differentiated high from low flavonoid consumers, in a multivariate setting. However, to confirm the superior performance of the Metabolite Fingerprint Score, further validation studies using external cohorts, different exposures, and different metabolomics platforms are required. The composition of the Metabolite Fingerprint Score provides insight into how systemic lipid metabolism differs across high and low flavonoid consumers.

## Supplementary material

For supplementary material accompanying this paper visit http://dx.doi.org/10.1017/jns.2017.27. click here to view supplementary material
