# Supplementary material for: Identifying the metabolomic fingerprint of high and low flavonoid consumers
Source: J Nutr Sci. 2017 Jul 14;6:e34. doi: 10.1017/jns.2017.27 (PMC5672306; doi:10.1017/jns.2017.27)
Supplement: Supplementary file 1 [file S2048679017000271sup001.doc]

**Supplementary Appendix S1:** Plasma metabolites excluded from analysis

To measure platform reproducibility, 6 plasma pools were randomly interspersed with case and control participant samples as blinded quality control samples. Coefficients of variance (CVs) were calculated for the plasma pools, and averaged to calculate the mean CV for each metabolite.

Based on the threshold of ≤ 25% for satisfactory reproducibility, 22 metabolites with a mean CV > 25% were excluded from our analyses. They were: indole-3-propionate; acetylcholine; glycerol; sucrose; uridine-5'-diphosphate (UDP); UDP-galactose/UDP-glucose; inosinic acid; cytidine; cytosine; cytidine monophosphate; gentisate; lactose; sarcosine; adenosine monophosphate; lysophosphatidylethanolamine C18:1; as well as the triacylglycerols C42:0, C56:3, C56:1, C44:0, C46:1, C56:10, C44:1.

Five metabolites that had undetectable levels for >10% of participants were also excluded. They were: 5-hydroxytryptophan; sebacate; benzoate; phosphocholine; suberate; phosphocreatine.

**Supplementary Appendix S2:** Plasma metabolites included in analysis

Following the exclusions, 174 metabolites were included in the analysis. These include: **diacylglycerols** (C34:2, C36:1, C34:1, C36:2); **triacylglycerols** (C46:0, C46:2, C48:0, C48:1, C48:2, C48:3, C48:4, C50:0, C50:1, C50:2, C50:3, C50:4, C50:5, C52:1, C52:2, C52:3, C52:4, C52:5, C52:6, C54:1, C54:2, C54:3, C54:4, C54:5, C54:6, C54:7, C54:8, C56:2, C56:4, C56:5, C56:6, C56:7, C56:8, C56:9, C58:10, C58:11, C58:7, C58:8, C58:9); **phosphatidylcholines** (C36:4A, C36:4B, C32:0, C34:1, C34:2, C34:3, C34:4, C32:1, C36:1, C32:2, C36:2, C36:3, C38:2, C38:3, C38:4, C38:5, C38:6, C40:6); **lysophosphatidylcholines** (C14:0, C16:1, C16:0, C18:2, C18:1, C18:0, C20:3, C20:4, C20:5, C22:6); **lysophosphatidylethanolamines** (C16:0, C18:0, C18:2, C20:4, C22:6); **sphingomyelins** (C14:0, C16:1, C18:0, C18:1, C18:2, C22:0, C16:0, C24:1); **cholesterol esters** (C16:1, C18:0, C18:2, C18:3, C20:5, C22:6, C20:3, C20:4); **bile acids** (chenodeoxycholate/deoxycholate, glycodeoxycholate/glycochenodeoxycholate, glycocholate, taurocholate, taurodeoxycholate/taurochenodeoxycholate); **amino acids** (glycine, alanine, arginine, serine, threonine, methionine, glutamate, asparagine, glutamine, histidine, lysine, valine, leucine, isoleucine, phenylalanine, tyrosine, tryptophan, proline, cis/trans hydroxyproline, ornithine, citrulline); **amino acid derivatives** (N-carbamoyl-beta-alanine, dimethyl-L-arginine/symmetrical dimethylarginine, N-monomethyl-L-arginine, aspartate, methionine sulfoxide, pyroglutamic acid, acetylglycine, dimethylglycine, 2-aminoadipate, kynurenine, kynurenic acid, anthranilic acid, 3-hydroxyanthranilic acid, quinolinate, xanthurenate, indoxylsulfate, thyroxine, aminoisobutyric acid); **amines** (phosphoethanolamine, putrescine, trimethylamine-N-oxide, choline, butyrobetaine, carnitine, acetylcarnitine, betaine, 1-methylnicotinamide); **organic acids** (lactate, pipecolic acid, citrate, aconitate, isocitrate, succinate, fumarate/maleate/alpha-ketoisovalerate, malate, 2-hydroxyglutarate, methylmalonate, 3-methyladipate/pimelate, adipate, alpha-hydroxybutyrate, beta-hydroxybutyrate); **vitamins** (pantothenate, 4-pyridoxate, thiamine); **other** (creatinine, creatine, allantoin, cotinine, hippurate, salicylurate, alpha-glycerophosphate, sorbitol, hydroxyphenylacetate, uracil, uridine, urate).
